# Prevalence and Molecular Characterization of Porcine Parvovirus 2 in Southwest China During 2020–2023

**DOI:** 10.3390/vetsci12020099

**Published:** 2025-01-30

**Authors:** Hongyu Chen, Yi Qing, Lei Xu, Ling Zhu, Wenqi Yin, Shuwei Li, Shengyao Kuang, Yuancheng Zhou, Zhiwen Xu

**Affiliations:** 1 Key Laboratory of Animal Diseases and Human Health of Sichuan Province, College of Veterinary Medicine, Sichuan Agricultural University, Chengdu 611130, China; chy58691498@outlook.com (H.C.); abtczl72@126.com (L.Z.); 2 National Center of Technology Innovation for Pigs, Chongqing 402460, China; 3 Chengdu Livestock and Poultry Genetic Resources Protection Center, Chengdu 610081, China; Qingy2024@163.com; 4 College of Animal Husbandry and Veterinary Medicine, Chengdu Agricultural College, Chengdu 610000, China; xu_lei2013@163.com; 5 Livestock and Poultry Biological Products Key Laboratory of Sichuan Province, Sichuan Animal Science Academy, Chengdu 610000, China; 411651624@scsaas.cn (W.Y.); lishuwei84511614@126.com (S.L.); ksy_cd@163.com (S.K.); 6 Animal Breeding and Genetics Key Laboratory of Sichuan Province, Sichuan Animal Science Academy, Chengdu 610000, China

**Keywords:** PPV2, high-throughput analysis, sow miscarriage, recombinant strain

## Abstract

Porcine parvovirus (PPV) is a non-enveloped, single-stranded linear DNA virus that induces reproductive disorders in sows, particularly abortions in primiparous sows. This study investigated the prevalence of PPV in the southwestern region and conducted molecular characterization of PPV strains. An epidemiological survey was conducted on 1534 aborted fetuses from the southwestern region between 2020 and 2023, revealing an abortion rate of 3.00% due to PPV2, with the highest rate of 3.77% in Sichuan. Additionally, 2973 blood samples from sows were tested using ELISA, showing a PPV2 antibody positivity rate of 73.03% to 90%. Through shotgun metagenomics, PPV2 SC2020 was identified in aborted fetal samples from a pig farm in Pengzhou, Sichuan. PCR sequencing analysis yielded seven PPV2 genomic sequences, and the phylogenetic analysis of eight PPV2 strains with thirty reference strains showed distinct evolutionary branches. The virus was successfully isolated from PPV2-positive samples, and the phylogenetic analysis of PPV2 SC2020 revealed ORF1 gene homology of 94.9% to 99.3% and the ORF2 gene homology of 93.1% to 98.0%, with 34 reference strains. Homologous recombination analysis indicated that SC2020 is a recombinant strain of HeB03 and S1.

## Simple Summary

The detection of porcine parvovirus 2 in aborted fetuses was achieved for the first time through the utilization of metagenomic sequencing. Notably, all sows on this farm were three-way hybrid sows, and upon delivering their second fetus, they exhibited a consistent occurrence of miscarriage, stillbirth, and mummified fetuses. Interestingly, porcine parvovirus type 2 was identified as the sole pathogen responsible for these abortions. Genetic evolution analysis revealed that the isolated porcine parvovirus 2 was a recombinant strain derived from the porcine parvovirus vaccine S1 strain and heb03 strain. After further investigation, it was found that the S1 strain of inactivated vaccine had been utilized, but cases of abortion caused by porcine parvovirus type 2 persisted.

## 1. Introduction

Porcine parvovirus (PPV) is a non-enveloped, single-stranded linear DNA virus with a genome of approximately 5 kb, which primarily induces reproductive disorders in sows, particularly leading to abortion in primiparous sows. The PPV virus is classified within the Parvoviridae family, specifically in the parvovirus subfamily and parvovirus genus. Since the initial report of PPV in 1967, a total of eight serotypes (PPV1–PPV8) have been identified in clinical cases [1]. PPV has the ability to infect all porcine species. If the maternal antibodies exist, the infection rate among piglets and weaned piglets will be lower than that of fattening pigs, wherein maternal antibodies play an important role [2]. At present, all PPV serotypes have been detected in China [3]. In 2001, PPV2 was accidentally discovered in pig sera from Myanma [4]. Ten years later, in China, it was found again on farms where pigs with “high fever disease” had a history of porcine respiratory and reproductive syndrome virus (PRRSV) and post-weaning multisystemic wasting disease (PMWS) [5]. Subsequently, porcine parvovirus 2 (PPV2) was detected successively in regions such as Europe, South Korea, Mexico, and other parts of the world [6,7,8]. In 2017, PPV2 was widely prevalent in Vietnam, with a detection rate as high as 61.6%. However, with the extensive promotion and application of relevant vaccines, PPV2 has been effectively controlled. By 2021, the detection rate of PPV2 had dropped to 3.4% [9]. From 2015 to 2017, the proportion of suckling piglets infected with PPV2 in Poland showed a remarkable trend, with the positive rate unexpectedly exceeding the crucial figure of 50% [2]. The PPV2 genome has a total length of approximately 5.4 kb [5] and contains two open reading frames: ORF1 encodes a nonstructural protein, while ORF2 encodes a structural protein. The prevalence of PPV2 is global, with a 40% infection rate observed in China. This high infection rate leads to frequent recombination events [10]. Recombination events of the capsid protein (VP) gene of porcine parvovirus type 2 (PPV2) have been reported. These events likely involve intra- and interspecies recombinations, as well as recombinations within and between strains from different countries. Moreover, the observed radiation pattern in the diversification of PPV2 strains indicates that genetic flow has taken place [8], thereby increasing the pressure on the prevention and control of PPV2. From 2018 to 2020, large-scale sow abortions in a Sichuan pig farm caused huge economic losses. In early 2020, our team identified porcine parvovirus 2 (PPV2) as the cause via metagenomic sequencing. After re-immunization and antibody tests, the abortion rate dropped significantly. Realizing PPV2’s key impact, this study investigated the prevalence of PPV in the southwestern region and conducted molecular characterization of the PPV strains.

## 2. Materials and Methods

### 2.1. Sample Collection and PCR Identification

From 2020 to 2023, employing a risk-based sampling (RBS) approach, we collected 1534 aborted fetuses and 2973 blood samples from a total of 69 pig farms in the southwestern region of China, including Guizhou Province, Chongqing Municipality, Yunnan Province, and Sichuan Province [9].

Aborted fetal lungs were collected and mixed with phosphate-buffered saline (PBS), then placed in sterile 1.5 mL microcentrifuge tubes containing steel balls. The mixture was homogenized using a Scientz-48l high-throughput tissue homogenizer (Ningbo Scientz Biotechnology Co., Ltd., Ningbo, China) at 4 °C and 50 Hz, for 60 s. The homogenate underwent three freeze–thaw cycles and was then centrifuged at 12,000× *g*. The resulting supernatant was immediately used for DNA extraction or stored at −80 °C for future use. DNA extraction was performed using a universal genomic DNA kit (Cofitt, Yancheng China), following the manufacturer’s instructions. For the detection of PPV2 in clinical samples, a SYBR Green I-based real-time PCR detection method was employed using the following primers: VP1 forward primer (5′-ATGAGCGCTGCCGACGCGTG-3′) and VP1 reverse primer (5′-CATTGATCCCTCTCCGCCCG-3′). Serum was extracted from the blood samples for ELISA analysis. The PPV2 antibody level was evaluated by the Chinreal commercial ELISA kit (Chinreal, Shengzhen, China).

### 2.2. DNA Extraction, Library Construction, and Sequencing

First, 200 milligrams of tissue was ground in 500 microliters of sterile water free of RNase and DNase. Subsequently, 200 microliters of the resulting lung homogenate was processed using the commercial QIAamp DNA Mini Kit (Qiagen, Hilden, Germany) for nuclease treatment, strictly following the manufacturer’s instructions to extract DNA. To determine the concentration of DNA, a biophotometer was used to measure the quality values of the viral DNA (A260/280 and A260/230). Subsequently, a Qubit 3.0 Fluorometer (Life Technologies, Carlsbad, CA, USA) with the dsDNA HS (High Sensitivity) Assay Kit (Invitrogen, Waltham, MA, USA) was employed to obtain a more accurate quantification, according to the manufacturer’s instructions.

The Illumina Sequencing workflow can be divided into four separate processes: library preparation, cluster generation, sequencing, and data analysis. Briefly, viral DNA was fragmented using NEBNext^®^ dsDNA Fragmentase^®^(NEB, Ipswich, MA, USA) and the resulting fragments were utilized in the NEB “NEBNext^®^ Ultra™ II DNA Library Prep Kit for Illumina^®^” (NEB, Ipswich, MA, USA) protocol. An Agilent 2100 Bioanalyzer with a high-sensitivity DNA kit was used to quantify the library. Paired-end 150 nt reads were generated according to the Illumina protocol “Prepare DNA libraries for sequencing on the NovaSeq 6000”.

### 2.3. RNA Extraction, Library Construction, and Sequencing

The RNA was extracted from samples of clarified infected cell lysate or purified virus using TRIzol, and prepared for next-generation sequencing. Briefly, reverse transcription was performed using random hexamers. Subsequent DNase treatment and cleanup were carried out, followed by second-strand synthesis before library preparation using Nextera XT reagents (Illumina, San Diego, CA, USA) and sequencing on the NovaSeq 6000 (Illumina). Although originally described as a consensus-level sequencing methodology, the depth of coverage allowed for deep sequencing analysis as well. Bioinformatics analysis of the data was completed using the pipeline previously described.

### 2.4. NGS Read Processing and Sequence Assembly

The read quality trimming of the reads was performed using the Skewer, with an additional trimming filter step to remove unreliable sequences based on a user-specified quality score [11]. Host read subtraction by read mapping was performed using the bwasw program (version 0.7.9a-r786) against ribosomal RNAs (16S, 18S, 23S, 28S, 5S), bacterial genome sequences, and the latest host organism genome sequences [12]. The de novo assembly followed the A5-miseq pipeline [13]. Finally, the scaffolds were subjected to bwasw read mapping and a mega-blast homology search against the NCBI NT database.

### 2.5. Virus Isolation

Porcine parvovirus 2-positive tissue homogenates were centrifuged at 12,000 rpm for 10 min at 4 °C. The resulting supernatant was then filtered through a 0.22 μm filter (Thermo Scientific, Waltham, MA, USA). The final filtrates were inoculated

ST cells and incubated in a 37 °C incubator supplemented with 5% CO_2_. After incubating at 37 °C for 1 h, the supernatant was removed, and the cells were cultured in DMEM supplemented with 2% FBS for 72 h. Daily observation was carried out to monitor the cytopathic effects (CPEs). The infected cells were fixed with 4% paraformaldehyde, and characterized using an immunofluorescence assay (IFA) employing an anti-PPV2 VP2 protein polyclonal antibody that had been prepared and stored in our laboratory.

### 2.6. Phylogenetic Analysis

Total genomic DNA from the positive samples was extracted utilizing a universal genomic DNA extraction kit from Cofitt (Yancheng, China). PCR amplifications were carried out employing PrimeSTAR DNA Polymerase (TaKaRa Bio, Shiga, Japan), with gene-specific primers, as detailed in Table S1. The purified PCR products, confirming the presence of the target genes, were cloned into the pUCm-T vector. Subsequently, these clones were dispatched to Shanghai Bioengineering Co., Ltd (Shanghai, China). for Sanger sequencing services. The alignment of the obtained full-length sequences was performed using DNAStar version 15.7 software to ensure accuracy and consistency. For the construction of phylogenetic trees, Molecular Evolutionary Genetics Analysis (MEGA; version 5.1) software was utilized, applying the maximum likelihood method and substantiated with 1000 bootstrap replicates to assess the robustness of the phylogenetic inferences. This rigorous approach facilitated a comprehensive genetic analysis and accurate phylogenetic placement of the samples within the context of known genetic lineages.

### 2.7. Recombination Analyses

The sequences of the PPV2 were identified in this study and 30 reference sequences from Genbank for recombination screening. We used SIMPLOT v3.5.1 to check the recombination signals and estimate the breakpoint locations. Furthermore, the recombination events were confirmed with a neighbor-joining phylogenetic tree.

## 3. Results

### 3.1. Epidemiological Survey of PPV2

**Figure 1:**
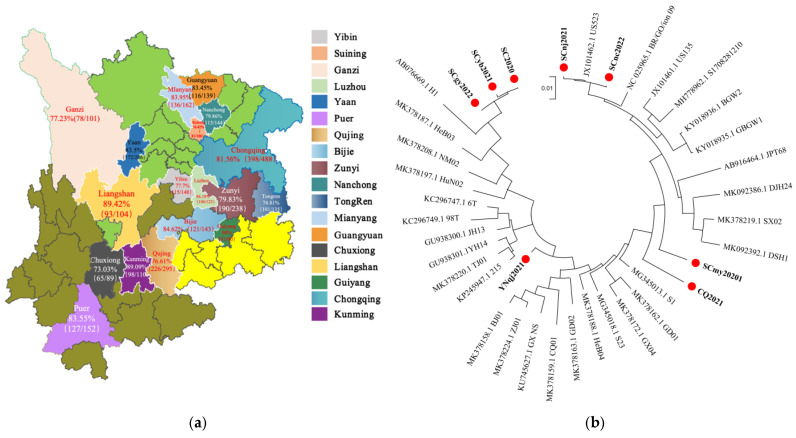
The epidemiological study of porcine parvovirus 2 in the southwestern region of China. (**a**) A serological investigation of PPV2; (**b**) a phylogenetic analysis of the full genome of PPV2.

The commercial pig farm located in Pengzhou, Sichuan Province has been confronted with a series of sow abortions since 2018. By employing shotgun metagenomics, we successfully identified PPV2 as the sole infectious agent present in the aborted fetal samples collected from this facility. The sequence assembly of the detected PPV2 yielded a 5179 bp contig, encompassing 95% of the genome sequence, with both ORF1 and ORF2 regions remaining intact. This strain was designated as PPV2 SC2020. Our subsequent epidemiological investigation into the prevalence of PPV2 in the southwestern region spanned 2020 to 2023, during which we conducted an analysis of a total of 1534 aborted fetuses. Our findings revealed that the abortion rate associated with PPV2 was 3.00% (46/1534) (Table 1). Notably, the rate in Sichuan Province was higher, at 3.77% (34/902), followed by Guizhou Province at 2.81% (7/249), Chongqing Municipality at 1.51% (1/66), and Yunnan Province at 1.26% (4/317) (Table 1). On farms testing positive for PPV2, the abortion rate was found to be at least 5.74%, with a maximum of 18.46% (Table 2). Furthermore, a total of 2973 blood samples were collected from sows across the southwestern region of China between 2020 and 2023. The ELISA test results (Supplementary Table S1) demonstrated that the prevalence of PPV2 antibodies in southwest China ranged from 73.03% to 90% (Figure 1a), indicating a generally satisfactory level of immunity against PPV2. However, as can be clearly seen from the etiological and serological data, although the positive rate of PPV2 antibodies is relatively high, the detection rate of PPV2 nucleic acid and the abortion rate on pig farms are not at a low level.

To elucidate the genomic characteristics of the prevalent PPV2 virus in the southwestern region, we undertook PCR sequencing analysis on positive samples and successfully obtained seven genomic sequences of PPV2. This study conducted a phylogenetic analysis of the genetic sequences of PPV2, comparing them with thirty reference strains. Based on comprehensive genomic sequence comparison, the SCnj2021 and SCnc2022 strains were found to cluster together with the reference strain US523 on a single phylogenetic branch. The SCmy2020 and CQ2021 strains were identified to be closely related, forming their own distinct branch separate from other strains. Notably, the YNqj2021 strain exhibited a more divergent evolutionary trajectory compared to the SCgy2022, SCyb2021, and SC2020 strains, which demonstrated a closer phylogenetic relationship by clustering with the H1 strain on a common branch (Figure 1b).

### 3.2. Viral Isolation

**Figure 2:**
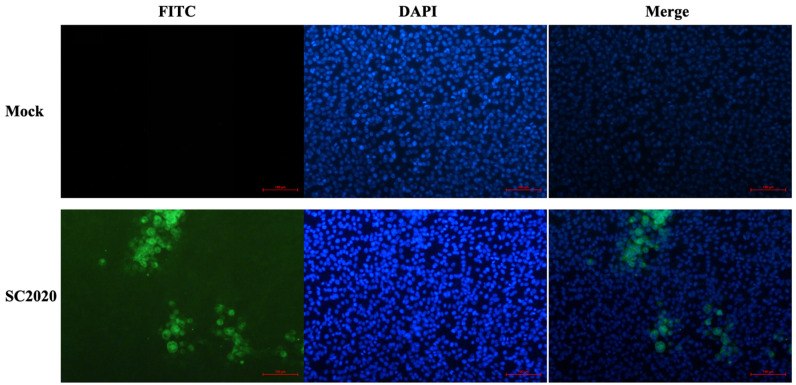
IFA of the PPV2 SC2020 strain. 4′,6-diamidino-2-phenylindole (DAPI): DAPI was utilized for staining cell nuclei. Fluorescein isothiocyanate (FITC): following the binding of the anti-VP2 protein mouse polyclonal antibody to the PPV2 VP2 protein, FITC-labeled goat anti-mouse immunoglobulin G (IgG) was utilized to bind to the complex, resulting in the emission of green fluorescence. Merge: combine DAPI and FITC staining pictures.

After grinding and filtering the collected PPV2-positive lung samples, the supernatant containing the virus was used to infect PK-15 cells, which were subsequently blindly passaged for five generations. Further confirmation was achieved through an immunofluorescence assay, where a pronounced green fluorescent signal was detected in cells inoculated with PPV2 SC2020, in contrast to the control group, for which no green fluorescent signal was observed (Figure 2). Collectively, these results demonstrate the successful isolation and characterization of the PPV2 virus strain.

### 3.3. Phylogenetic Analysis of SC2020

**Figure 3:**
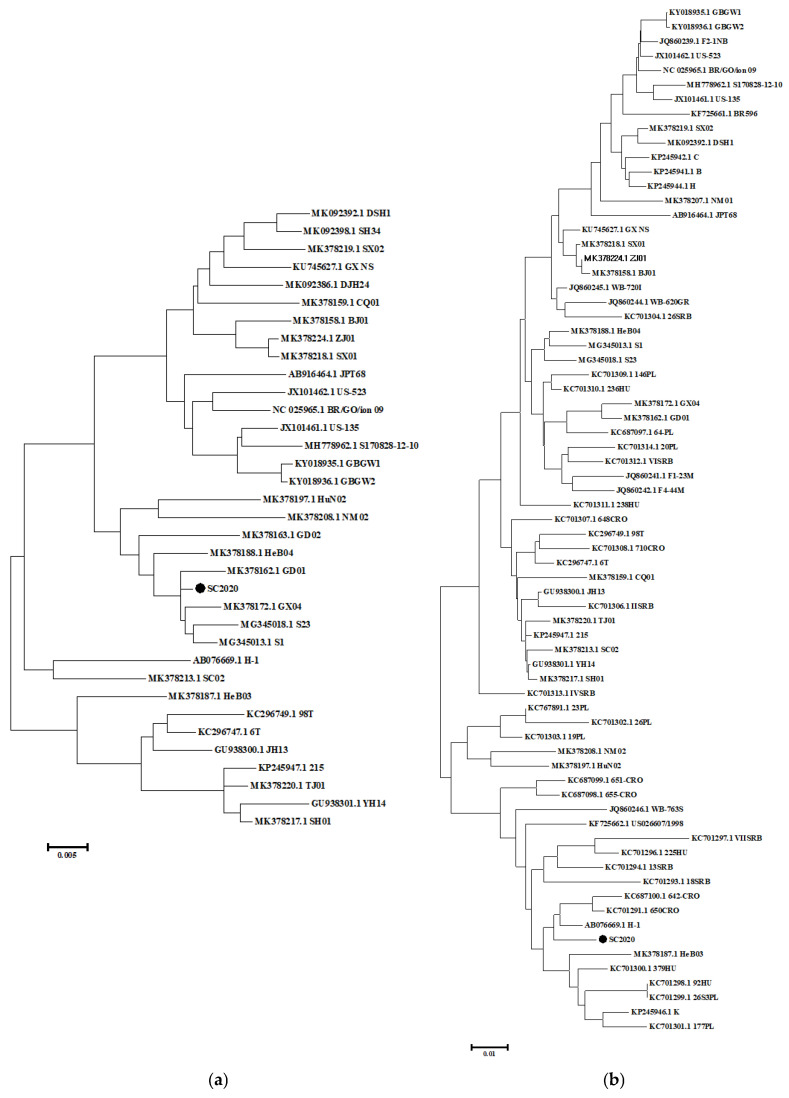
Phylogenetic tree analysis of the PPV2 SC2020 strain. The reliability of the tree was evaluated using the maximum likelihood method, employing the Tamura Nei model and performing 1000 bootstrap repeats. (**a**) Phylogenetic analysis was performed based on the PPV2 ORF1 gene; (**b**) phylogenetic analysis was conducted based on the PPV2 ORF2 gene.

A comprehensive analysis of the genetic sequence of PPV2 SC2020 was conducted to elucidate its characteristics and potential implications for the disease. Briefly, the ORF1 gene sequences of 34 PPV2 reference strains obtained from NCBI were used for phylogenetic analysis. The homology of the ORF1 gene with 34 reference strains ranged from 94.9% to 99.3%. The highest degree of similarity was observed with the S1 strain (MG345013.1) and the GX04 strain (99.3%), while the lowest was with the 215 strain (KP245947.1) (94.9%) (Figure 3a and Figure S1). The homology of the ORF2 gene with these sequences ranged from 93.1% to 98.0%; the highest level of similarity was 98.0% (AB076669.1) with the H-1 strain, while the lowest level recorded was 93.1% (KC701306.1) with the IISRB strain (Figure 3b and Figure S2).

### 3.4. Homologous Recombination Analysis

**Figure 4:**
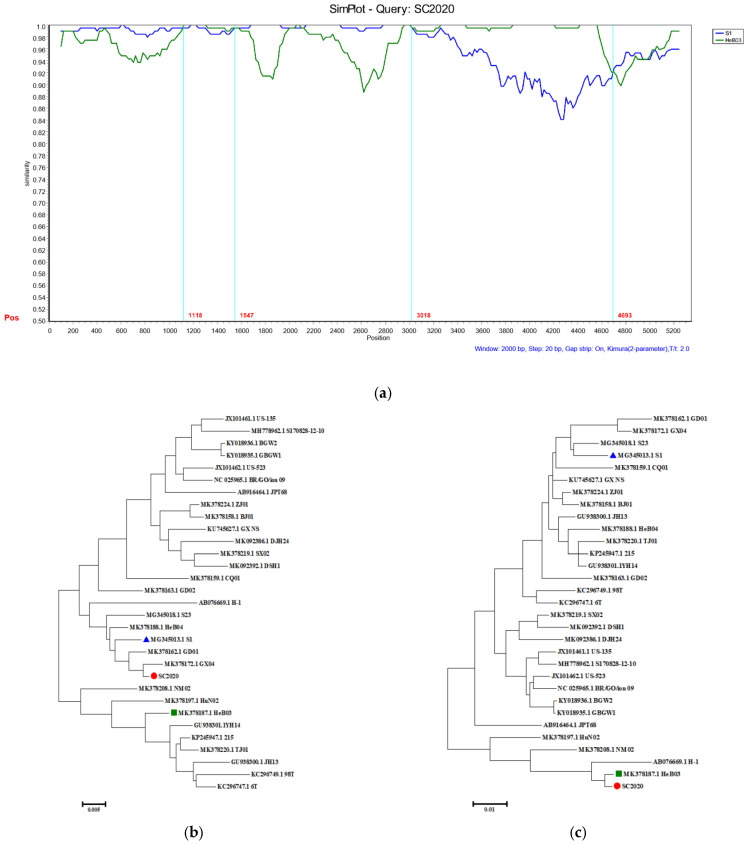
Recombination analysis of the PPV2 SC2020 genome. (**a**) The genetic similarity between the recombinant strain and its parental strain was assessed using SimPlot. The PPV2 SC2020 genome served as the query sequence. Recombination breakpoints were denoted by red lines, with their position indicated at the bottom. (**b**) The phylogenetic analysis was conducted based on nucleotide (NT) regions 1–1117 nt, 1548–3017 nt, and 4694–5179 nt. (**c**) The phylogenetic analysis was conducted based on nucleotide (NT) regions 1118–1547 nt and 3018–4693 nt.

All recombination events were detected and analyzed using SimPlot, followed by the construction of a basic phylogenetic tree. The sequence alignment revealed the presence of two recombination breakpoints (Figure 4a). The SC2020 strain exhibited a high degree of similarity to the HeB03 strain in both the 1118–1547 nt region and the 3018–4693 nt region, encompassing segments of the nonstructural protein and structural protein. We observed a high degree of similarity between SC2020 and S1 in the regions spanning nucleotides 1–1117, 1548–3017, and 4694–5179. The phylogenetic analysis showed that the 1118–1547 nt and the 3018–4693 nt regions of SC2020 were clustered with the HeB03 strain (Figure 4b), while the 1–1117 nt, 1548–3017 nt, and 4694–5179 nt regions were associated with the S1 strain (Figure 4c).

## 4. Discussion

Parvovirus type 2 primarily infects sows, resulting in reproductive dysfunction characterized by abortion, stillbirths, abnormal fetuses, mummified fetuses, and compromised neonatal health in primiparous sows; however, affected sows do not exhibit evident clinical symptoms. PPV2 is mainly localized in lung lymphocytes, resulting in a significant narrowing of the alveolar space, vascular congestion, infiltration of lymphocytes, and necrosis of ciliary epithelial cells [14]. Therefore, the detection of PPV2 is commonly conducted in lung tissue samples obtained from dead pigs. Currently, reports have confirmed the detection of PPV2 in blood, feces, pleural effusion, and lung tissue of piglets [15]. The isolation and identification process is commonly performed using lung tissue samples obtained from piglets or fattening pigs [6]. Additionally, the transmission of PPV from sow to piglet can occur through the placental barrier. It is possible that many researchers focus on dead pigs and sows while disregarding aborted fetuses. Multiple PPV viruses, including PPV2, have been detected in the hearts of aborted fetuses. However, the most prevalent ones are PPV4, PPV6, and PPV7 [7]. Our findings align with this characteristic.

PPV2 is susceptible to co-infection with PCV and PRRSV, which can cause respiratory symptoms and reproductive disorders [16]. It was observed that in the co-infection cases of novel PPV and PCV, PCV and PPV2 were the most common [17]. Porcine parvovirus (PPV) has been found to be prevalent in several countries such as Vietnam, South Korea, Italy, Poland, and Serbia [18,19,20,21]. In South Korea, the prevalence of porcine parvovirus (PPV) shows a diverse trend. PPV1, PPV2, PPV6, and PPV7 have become the main prevalent virus strains. Among them, the detection rate of PPV2 is quite remarkable, reaching 22.1%. Closely following is PPV6, with a detection rate of up to 21.5%. Moreover, the situation of mixed viral infections on South Korean pig farms is relatively common. In samples positive for porcine circovirus type 2 (PCV2), the detection probability of PPV1 and PPV6 is higher. In samples positive for porcine reproductive and respiratory syndrome virus (PRRSV), the positive rates of PPV2 and PPV7 are more prominent [19]. In Serbia, the prevalent PPV strains include PPV1, PPV2, and PPV3. In the detection of porcine parvovirus (PPV) in the northern region of Italy, the detection rates of PPV2 and PPV3 are relatively low, being 2.4% and 3.2%, respectively. Conversely, the detection rates of PPV1, PPV5, and PPV6 are relatively high, reaching 31.5%, 24.2%, and 22.6%, successively. Moreover, the phenomenon of the co-infection of porcine parvovirus with porcine circovirus type 2 (PCV2), porcine circovirus type 3 (PCV3), and porcine reproductive and respiratory syndrome virus type 1 (PRRSV-1) is relatively prominent in the local pig population [22]. Notably, in southern Italy, the detection results of wild boar reproductive tissues show that the positive rate of PPV is as high as 44.4% [21].

PPV2 and PPV6 have a high prevalence in the hearts of fetuses. In 2009, Rinku Sharma reported the detection of PPV2 in dead piglets within 7 days of age. Moreover, the isolated strain of PPV2 was also found in the tissues of finishing pigs. The majority of the PPV-positive pigs were found to be co-infected with two or three different types of viruses. The co-infection of PPV2 and PPV3 was found to be the predominant occurrence in domestic pigs, while single infection was observed as the prevailing type in wild boars [10]. The present study revealed that a single PPV2 infection, as identified through shotgun metagenomics, was responsible for the occurrence of abortion. It is noteworthy that PPV2 exhibits a high propensity to induce miscarriage in primipara sows. After immunization, the sows exhibited effective protection. However, during their second birth, all sows experienced miscarriages, stillbirths, and mummified fetus despite being vaccinated with the S1 strain of inactivated vaccine. This indicates that the protection provided by the S1 vaccine is insufficient.

The phenomenon of PPV2 recombinant is highly prevalent. Regarding the recombination of PPV (Porcine Parvovirus), the academic community generally believes it is closely linked to the characteristics of the pig production system. For instance, the pig population density is too high; on some pig farms, the immunization programs are imperfect, leading to uneven herd immunity, which makes it easier for the virus to mutate and recombine in susceptible individuals. These factors interact with each other, intensifying the complexity of PPV recombination [23]. In a study conducted by Dániel Cadar et al., nine strains of PPV2 were documented [10]. Recombination analysis showed that the SC2020 strain resulted from a fusion between the S1 and HeB03 strains. This discovery is of great importance for understanding the genetic diversity of PPV2. In summary, the emergence of novel recombinant PPV2 strains poses significant challenges for preventing and controlling PPV2.

## Figures and Tables

**Table 1:** Prevalence of PPV2 in aborting sows in Southwest China from April 2020 to September 2023.

Province	Region	No. Sample	No. Positive	Positive Percentage
Sichuan 3.77% (34/902)	Yibin	96	6	6.25%
	Nanchong	97	7	7.22%
	Guangyuan	50	1	2.00%
	Bazhong	53	0	0.00%
	Mianyang	54	2	3.70%
	Chengdu	63	5	7.94%
	Deyang	92	2	2.17%
	Neijiang	48	1	2.08%
	Meishan	78	0	0.00%
	Yaan	70	4	5.71%
	Leshan	85	6	7.06%
	Luzhou	44	0	0.00%
	Dazhou	72	0	0.00%
Chongqing 1.51% (1/66)	Chongqing	66	1	1.52%
Yunnan 1.26% (4/317)	Zhaotong	80	0	0.00%
	Kunming	54	2	3.70%
	Qujing	71	1	1.41%
	Baoshan	70	0	0.00%
	Puer	42	1	2.38%
Guizhou 2.81% (7/249)	Guiyang	58	0	0.00%
	Anshun	57	0	0.00%
	Zunyi	63	1	1.59%
	Tongren	71	6	8.45%
Total	Southwestern of China	1534	46	3.00%

**Table 2:** The abortion rate of sows on pig farms testing positive for PPV2.

Province	Region	Farm	Batch Abortion Rate
Sichuan	Yibin	Pinshan #1	10.34%
		Junlian #2	15.18%
		Jiangan #3	15.36%
	Nanchong	Yingshan #4	16.41%
		Liangzhong #5	12.61%
		Yilong #6	15.65%
		Nanchong #7	11.42%
	Guangyuan	Wangchang #9	8.34%
	Mianyang	Santai #1	11.63%
	Chengdu	Chongzhou #17	18.46%
		Meishan #18	9.33%
		Xinjing #20	15.76%
	Deyang	Mianzhu #21	5.74%
	Neijiang	Zizhong #24	8.65%
		Longchang #25	14.88%
	Yaan	Yaan #28	17.84%
		Yaan #30	17.72%
	Leshan	Leshan #33	11.48%
Chongqi	Chongqing	Rongchang #42	10.40%
Yunnan	Kunming	Kunming #46	18.24%
	Qujing	Xuanwei #48	8.27%
	Puer	Puer #54	16.81%
Guizhou	Zunyi	Tongzhi #63	8.35%
	Tongren	Tongren #69	17.20%

## Data Availability

No new data were created or analyzed in this study. Data sharing is not applicable to this article.

## Supplementary Materials

The following supporting information can be downloaded at: https://www.mdpi.com/article/10.3390/vetsci12020099/s1, Figure S1: Homology analysis of PPV2 ORF1 gene; Figure S2: Homology 236 analysis of PPV2 ORF2 gene; Table S1: Seroprevalence of PPV2 in serum samples of 237 pigs in Southwest China.
