# Supplementary material for: Prevalence and Molecular Characterization of Porcine Parvovirus 2 in Southwest China During 2020–2023
Source: Vet Sci. 2025 Jan 30;12(2):99. doi: 10.3390/vetsci12020099 (PMC11861861; doi:10.3390/vetsci12020099)
Supplement: Supplementary file 1 [file vetsci-12-00099-s001.zip › Supplementary Table S1.pdf]

Supplementary Table S1. Seroprevalence of PPV2 in serum samples of pigs in Southwest China

Province	Region	2020		2021		2022		2023		Positive Percentage
		No. sample	No. positive	No. sample	No. positive	No. sample	No. positive	No. sample	No. positive	
Sichuan	Yibin	20	14	35	24	52	44	41	33	77.70%
	Nanchong	40	31	41	31	23	18	40	35	79.86%
	Suining	22	13	12	7	44	38	28	23	76.42%
	Ganzi	18	15	13	9	43	32	27	22	77.23%
	Mianyang	2	2	37	33	68	49	55	52	83.95%
	Luzhou	17	12	44	42	33	26	29	26	86.18%
	Guangyuan	50	39	46	39	19	14	24	24	83.45%
	Yaan	76	61	41	34	50	44	39	33	83.50%
	Liangshan	21	17	27	26	25	21	31	29	89.42%
Chongqing	Rongchang	26	15	40	33	133	113	94	84	83.62%
	Wanzhou	31	23	51	39	52	42	61	49	78.46%
Yunnan	Qujing	77	48	75	56	78	64	65	58	76.61%
	Chuxiong	11	7	15	10	38	24	25	24	73.03%
	Puer	30	19	21	21	57	45	44	42	83.55%
	Kunming	19	14	19	17	29	27	43	40	89.09%
Guizhou	Tongren	12	6	35	22	23	14	65	59	74.81%
	Bijie	13	8	14	11	60	51	56	51	84.62%
	Zunyi	42	26	59	48	67	58	70	58	79.83%
	Guiyang	10	8	12	12	31	27	37	34	90.00%
Total		537	378	637	514	925	751	874	776	81.37%
